# Effects of stretching on menopausal and depressive symptoms in middle-aged women: a randomized controlled trial

**DOI:** 10.1097/GME.0000000000000651

**Published:** 2016-08-05

**Authors:** Yuko Kai, Toshiya Nagamatsu, Yoshinori Kitabatake, Hiroomi Sensui

**Affiliations:** 1 Physical Fitness Research Institute, Meiji Yasuda Life Foundation of Health and Welfare, Tokyo, Japan; 2 Department of Health Sciences, Saitama Prefectural University, Saitama, Japan.

**Keywords:** Climacteric, Exercise, Occupational health, Physical activity, Women's Health Initiative

## Abstract

**Objective:**

Exercise may help alleviate menopausal and depressive symptoms in middle-aged women, but sufficient evidence does not currently exist to fully support this theory. Whereas frequent moderate- to vigorous-intensity exercise may be associated with the risk of menopausal hot flashes, light-intensity exercise, such as stretching, is not likely to increase the occurrence of hot flashes. Little is, however, known about the effects of light-intensity exercise on menopausal and depressive symptoms. We examined the effects of a 3-week stretching program on the menopausal and depressive symptoms in middle-aged, Japanese women.

**Methods:**

Forty Japanese women, aged 40 to 61 years, were recruited (mean age, 51.1 ± 7.3 y). The participants were randomly assigned to either a stretching or a control group. The stretching group (n = 20) participated in a 3-week intervention program that involved 10 minutes of daily stretching, just before bedtime. The control group (n = 20) was assigned to a waiting list. Menopausal symptoms were evaluated using the Simplified Menopausal Index, which measures vasomotor, psychological, and somatic symptoms. Depressive symptoms were assessed using the Self-Rating Depression Scale.

**Results:**

The compliance rate was 75.8% during the 3-week intervention program. The total Simplified Menopausal Index scores, including the vasomotor, psychological, and somatic symptoms, and the Self-Rating Depression Scale scores significantly decreased in the stretching group compared with that in the control group. No adverse events, including increased hot flashes, were reported by the participants during the study period.

**Conclusions:**

These findings suggest that 10 minutes of stretching before bedtime decreases menopausal and depressive symptoms in middle-aged, Japanese women.

Most women experience unpleasant symptoms during their menopausal transition. The most common menopausal symptoms are hot flashes, sleep disturbances, muscle or joint pains, and irritability.^1^ In addition, approximately 25% of perimenopausal women suffer from depressive symptoms.^2^ Hormone therapy (HT) is an effective treatment for such symptoms; however, many women seek other treatment with fewer adverse effects and health risks than those associated with HT.^3^ Evidence needs to be accumulated regarding possible lifestyle modification interventions that alleviate menopausal and depressive symptoms.

Exercise may help alleviate menopausal and depressive symptoms among middle-aged women, but insufficient evidence currently exists to support this theory.^4^ Aerobic exercise training for 6 months has been reported to improve menopausal symptoms^5^ and quality of life.^6^ Furthermore, a prospective study showed a relationship between moderate- to vigorous-intensity exercise and depressive symptoms in middle-aged women.^7^ In a review of randomized controlled trials (RCTs) involving adults with depression, the effect size of exercise on depression was −0.62 compared with that in the control group, indicating a moderate clinical effect.^8^ Although the data on the effects of exercise in perimenopausal women have been equivocal, recent reviews clearly point to the need for more evidence based on RCTs.^4,9^ In addition, most of the previous studies focused on the effects of moderate- to vigorous-intensity exercise, which may actually trigger hot flashes in these women.^10^ Light-intensity exercise is not likely to increase the occurrence of hot flashes, but has a positive impact on psychological well-being.^11,12^ Little is, however, known about the effects of light-intensity exercise on menopausal and depressive symptoms in middle-aged women.^13,14^

Light-intensity exercise is defined as less than three metabolic equivalents (METs).^15^ One common, light-intensity exercise is stretching, which is regarded as providing approximately 2.3 METs.^16^ Previous reports have indicated that daily stretching for 15 minutes suppresses sympathetic nervous activity and increases parasympathetic activity.^17^ The basic causes of physical and mental health-related complaints in perimenopausal women seem to be related to a disorder of the autonomic nervous system, in addition to a decline in estrogen levels.^4^ Therefore, stretching may help to improve both physical and mental health of perimenopausal women, but evidence in this regard remains unclear. The purpose of this study was to examine the effects of stretching on menopausal and depressive symptoms in middle-aged women.

## METHODS

### Study setting and participants

This single-center, two-armed, parallel-group, RCT was part of a sleep study in middle-aged, Japanese working women. The study participants were sales or clerical staff at a life insurance company in Tokyo, Japan, recruited via flyers distributed at their worksite. The eligibility criteria included (1) less than 40 years of age; (2) less than 1 point on the Simplified Menopausal Index (SMI, explained below); (3) no limitations on exercising (orthopedic disorders); (4) no current therapies such as HT, psychotropic medications, or sleeping pills; and (5) no history of surgical menopause. The study participants were stratified by age group (40-49 and >50 y), and the members of each age group were randomly assigned to the stretching and control groups. The randomization was centralized and performed on the basis of a computer-generated list of random numbers. Staff, independent of the investigators, informed the participants about the details of their allocated group. The stretching group participated in a 3-week stretching intervention program, whereas the control group was assigned to a waiting list.

All procedures followed were in accordance with the ethical standards of the responsible committee on human experimentation (institutional and national) and with the Helsinki Declaration of 1975, as revised in 2000. Signed informed consent was obtained from all participants being included in the study. This study was approved by the ethics committee of the Physical Fitness Research Institute, Meiji Yasuda Life Foundation of Health and Welfare, Tokyo, Japan.

### Study protocol

The baseline survey assessed menopausal and depressive symptoms and baseline characteristics of each of the participants. In the stretching group, the 3-week stretching intervention program began 1 week after the baseline survey. After the intervention, the participants of both groups were resurveyed regarding menopausal and depressive symptoms.

### Measurements

Menopausal symptoms were assessed using the SMI, which comprised 10 questions assessing vasomotor (4 items; hot flashes, chills, etc), psychological (4 items; mood, sleep disturbances, etc), and somatic (2 items; joint pain, shoulder stiffness) symptoms.^18^ The SMI is a survey created to assess menopausal symptoms of Japanese women; this index is frequently used for research and during routine hospital examinations in Japan.^19^ The frequency of hot flashes was calculated based on the number of participants experiencing sudden feelings of facial heat.

Depressive symptoms were assessed using the Self-Rating Depression Scale (SDS),^20^ which is a self-assessment questionnaire that assesses depressive symptoms, and is internationally used for research. Depression was considered present if the SDS score was 40 points or more.

The basic characteristics of the participants, including age, alcohol consumption, smoking habits, job strain score,^21^ and total weekly leisure-time moderate- to vigorous-intensity physical activities, were examined by a self-reporting questionnaire. Menopausal status was assessed by asking questions related to menstruation. Postmenopausal women were defined as those women without menstrual bleeding during the previous 12 months.^1^

### Intervention program

The 3-week intervention comprised home-based, primary stretching sessions and group-based, ancillary sessions. The primary sessions consisted of a 10-minute stretching program that was performed once a day at home. The stretching program was designed to be performed just before bedtime to relax and to induce sleep, and to promote the practice and continuation of the stretching program, even if the participants lacked time to exercise during the day. The basics of this stretching program involved total body extensions, including extension and torsion of the shoulders, hips, and torso. The stretching program was designed to start with standing (or kneeling) poses, move to sitting poses followed by poses in the prone position, and end in a relaxed supine position. Simple yoga poses were referenced when developing some of the stretches. The stretches comprising the daily stretching program were changed weekly, except for the relaxed supine position that ended the program. The group-based ancillary sessions were held weekly for the stretching group participants at their work site, and the stretching program for the following week was explained during this 30-minute session. A leaflet explaining the week's stretching program and a self-monitoring worksheet were also distributed during each group session. The participants were directed to keep a record of their practice by filling out a daily self-monitoring sheet, and the average number of days per week that the stretching program was implemented at home was obtained from these records. During the study period, the research staff did not introduce the participants to other healthcare services, nor did they recommend any self-care methods other than stretching.

### Sample size calculation

The estimated standard deviation of the total SMI score postintervention was 15 points, as estimated in a previous study.^22^ Based on this standard deviation, a type 1 error probability of 5%, and a power of 80%, we determined that 16 participants should be included in each group to detect a minimum relevant difference of 15 points on the SMI scale. Anticipating unavailability of 20%, the present study aimed to enroll 20 participants to offset the potential loss of power.

### Statistical analyses

Data analyses were conducted using intention-to-treat by inputting baseline values forward for the dropout participant. Characteristics were compared using an unpaired *t* test for continuous data, and a χ^2^ analysis for categorical data. Intervention effects were evaluated using generalized linear models, adjusted for job strain scores and initial values. Moreover, Cohen's *d* effect sizes were calculated using the differences in the change between both groups (stretching group minus control group). *P* < 0.05 was considered statistically significant. All analyses were performed using SPSS version 21 (SPSS Japan, Tokyo, Japan).

## RESULTS

**FIG. 1:**
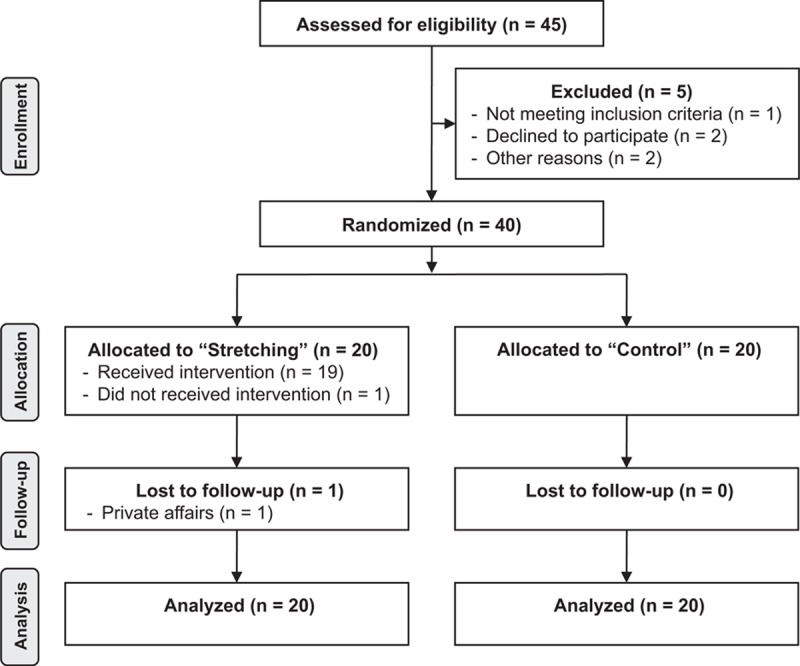
Flow diagram for the trial.

A total of 45 potential participants were recruited. Of these, one applicant was excluded on the basis of the above criteria, two declined to participate, and two were unable to participate (Fig. 1). Thus, in all, 40 participants were enrolled and randomized; 20 participants were allocated to the stretching group and 20 to the control group. Because of a change in personal circumstances, one participant in the stretching group dropped out of the study after the initial baseline survey, without receiving any intervention.

Over half of the participants were postmenopausal (55.0%) and had depression (62.5%). In addition, most of the participants were inactive and did not participate in leisure-time physical activity. A minority of the participants regularly consumed alcohol (40%) or smoked (27.5%). Significant differences in the average age, menopausal status, depression, hot flashes, alcohol and cigarette consumption, job stress, leisure-time physical activity, SMI scores, and SDS scores were not observed between the groups at baseline (Table 1).

The average number of days per week that the stretching program was implemented at home was 5.1 ± 1.9 days for the first week, 5.4 ± 1.8 days for the second week, and 5.4 ± 1.9 days for the third week. The overall implementation rate for the 3-week intervention program was 75.8%.

The total SMI scores (effect size = −0.86) including the vasomotor (−0.78), psychological (−0.48), and somatic (−0.84) symptoms, and the SDS (−0.46) scores significantly decreased in the stretching group compared with that in the control group (Table 2). Five of the 12 participants (41.7%) who had depression at baseline recovered to the normal level after the intervention in the stretching group. In contrast, only 2 of the 13 participants (15.4%) recovered in the control group.

The frequency of hot flashes after the intervention was not significantly different between the stretching (25%) and control (45%) groups (*P* = 0.320). No adverse events, including an increased frequency of hot flashes, were reported by any of the participants during the study period.

## DISCUSSION

In the present study, we examined the effects of a daily, 10-minute stretching routine over a 3-week period on the menopausal and depressive symptoms in menopausal, Japanese women. This study is the first RCT, to our knowledge, to show that stretching improves menopausal and depressive symptoms in middle-aged women. This study focused on the effects of light-intensity exercise, specifically stretching, on menopausal and depressive symptoms.

In contrast, current public health recommendations promote traditional aerobic exercise of moderate-to-vigorous intensity, such as walking or jogging.^23,24^ The stretching recommended before and after aerobic exercise is to prevent sports-related injuries. Therefore, there are fewer studies on light-intensity exercise than moderate- to vigorous-intensity exercise. In addition, the independent effects of stretching, in particular, have not been reported. Stretching has frequently been used as a control condition for exercise of moderate-to-vigorous intensity in previous studies.^25,26^ For example, Aiello et al^25^ held a weekly stretching session for the control group in an RCT involving postmenopausal women. They reported that neither moderate-intensity exercise nor stretching improved menopausal symptoms, including depressive feelings, and that moderate-intensity exercise, five times per week, slightly increased the incidence of severe hot flashes. Frequent exercise has also been often linked to an elevated risk of hot flashes among middle-aged women.^10^ The stretching program in their study^25^ was successful as a control condition, but may have been too infrequent to achieve the desired effect. The present study was designed for daily stretching and did not show any adverse events, including increased frequency of hot flashes, but significantly decreased the menopausal and depressive symptoms in the study participants. A large effect size (−0.84) was observed for somatic symptoms, in particular. This may have been due to improvements in muscle flexibility, contributing to the effect on somatic symptoms, including joint pain, shoulder stiffness, and others. These results suggest that stretching can be safely and frequently practiced by middle-aged women and is likely to improve menopausal and depressive symptoms.

Dunn et al^26^ compared aerobic exercise of moderate-to-vigorous intensity with a stretching-only control group in an RCT, targeting participants with depression. Their study concluded that aerobic exercise with an energy consumption equivalent to public health recommendations, rather than stretching, had a higher antidepressant effect; however, their study also showed that the control group, practicing a stretching routine three times a week, also reported a decrease in depressive scores by approximately 30% from the baseline. During the present study, the depressive score was reduced by approximately 20% from baseline in the stretching group, showing a statistically significant difference from the control group. The antidepressive effects of exercise have been validated, focusing on exercise of moderate-to-vigorous intensity, whereas the evidence for the benefits of light-intensity exercise is limited. A meta-analysis of the antidepressive effects of Tai Chi (1.5-3.0 METs),^16^ as an exercise of relatively light intensity, was investigated.^27^ Although the report indicated that a better study design was required, the effect size of Tai Chi was estimated to be 0.48, indicating a small-to-moderate effect. The effect size of stretching on depressive symptoms in the current study was similar. In terms of these results, both traditional aerobic exercise of moderate-to-vigorous intensity and light-intensity exercise may alleviate depressive symptoms.

The timing of the stretching may also be important for the present study. We directed the stretching group to practice stretching just before going to bed. A previous study^28^ reported that a brief stretching program, practiced just before bed, decreased sleep latency and contributed to better sleep. Perimenopausal women often complain of sleep problems,^29^ and an association between sleep and depressive symptoms has also been reported.^30^ In particular, difficulty in falling asleep was more strongly related to later depression.^31^ In the present study, the stretching performed before bedtime may have improved the participants’ sleep, leading to positive effects on menopausal and depressive symptoms.

Acute stretching suppresses sympathetic nervous activity and increases parasympathetic activity,^32^ which seems to be effective for achieving better sleep. In addition, daily stretching for 28 days can achieve similar chronic effects on the autonomic nerve system.^17^ The basic cause of physical and mental health complaints in perimenopausal women includes a complex association between physiological and psychosocial factors. Typical physiological factors are disorders of the autonomic nervous system, including sympathetic tone and/or parasympathetic inhibition, in addition to reduced estrogen levels.^4^ The effects observed in this study are presumed to involve changes in the autonomic nervous system, leading to improved sleep; however, the biological effects, such as changes in estrogen levels, stimulated by the stretching program applied in the current study were not shown in the perimenopausal women. Therefore, further studies are required to elucidate the mechanism by which stretching alleviates menopausal and depressive symptoms.

The present study targeted working women and may, therefore, be beneficial as a study of industrial health. The number of working women is increasing globally, but fewer studies have been conducted on their health, particularly of middle-aged women.^33^ The risk of depression is reportedly two times higher among women than among men,^34^ and work stress and other environmental factors exacerbate menopausal and depressive symptoms.^35^ Therefore, menopausal and depressive symptoms are important themes for health promotion in middle-aged working women. Although exercise seems to have mental and physical benefits, low levels of physical activity have been reported among women of menopause age.^36^ Studies have shown that the most strongly perceived barrier to exercise in women is their lack of time.^37^ Therefore, adherence to an exercise program by working women with insufficient time needs to be considered. In this study, adherence to the stretching program was encouraged; thus most participants could be performing the stretching routine for more than 5 days per week. This suggests that this stretching program could be implemented by busy women. As a result, the present industrial health study is able to propose a safe, concrete program for health promotion among middle-aged, working women.

As the present study did not involve a placebo control, it may overestimate the study results. As explained to the study participants and the intervention staff, who instructed to participants about the intervention program, this stretching program was an interventional procedure for improving sleep; therefore, the placebo effect on menopausal and depressive symptoms may be small. Nevertheless, the difference in the amount of staff contact between the stretching and control groups remains a problem. Because it is possible that contact with staff members may have a positive effect on mental health, we may have overestimated the effect of the stretching program. Future study of this issue should ensure that the same amount of staff contact is provided to both the stretching and control groups. The results of this study are limited because they targeted only a small number of Japanese women, and menopausal symptoms are known to be influenced by race and sociocultural differences.^1^ Further studies, involving different targeted groups of women, may be required to establish broader conclusions. Furthermore, additional follow-up surveys, conducted at regular intervals after the intervention program, may help to clarify the long-term effects of the stretching. Finally, we did not clarify the mechanisms underlying the stretching-improved menopausal and depressive symptoms. Therefore, further investigation is required to explore these mechanisms.

## CONCLUSIONS

We examined the effects of a 10-minute, daily stretching routine on menopausal and depressive symptoms in middle-aged, Japanese working women. The home-based stretching program was designed for performing just before bedtime. The results of this study suggest that 10 minutes of stretching decreased menopausal and depressive symptoms compared with women who did not receive this intervention; moreover, this stretching program did not increase the risk of hot flashes in these women.

## Figures and Tables

**TABLE 1:** Baseline characteristics of the study participants

	Stretching group (n = 20)	Control group (n = 20)	*P*^*a*^
Age, y	51.0 (7.0)	51.2 (7.9)	0.95
Postmenopausal women, %	55.0	55.0	1.00
Total SMI score (points)	29.3 (17.5)	32.4 (19.4)	0.61
Vasomotor symptoms	11.0 (7.3)	11.6 (8.5)	0.81
Physiologic symptoms	11.1 (10.4)	12.4 (10.8)	0.69
Somatic symptoms	7.3 (4.2)	8.4 (4.0)	0.42
SDS score (points)	40.1 (8.6)	42.8 (7.4)	0.30
Depression, %^*b*^	60.0	65.0	1.00
Hot flashes, %	20.0	30.0	0.72
Current drinker, %	40.0	40.0	1.00
Current smoker, %	30.0	25.0	1.00
Job strain score (points)^*c*^	0.48 (0.11)	0.43 (0.08)	0.13
Leisure-time physical activity, min/wk	29.3 (107.5)	34.8 (90.5)	0.86
<30 min/wk, %	90.0	80.0	0.66

Values are mean (SD), except categorical data. SMI, Simplified Menopausal Index; SDS, Self-Rating Depression Scale.

^*a*^*P* value by unpaired *t* test for continuous data, χ^2^ test for categorical data.

^*b*^Depression was defined with an SDS score ≥40 points.

^*c*^Job strain score was calculated using the Job Content Questionnaire.

**TABLE 2:** Menopausal and depressive symptoms at baseline (pre) and after the 3-week stretching program (post)

	Stretching group		Control group		Difference between groups	
	Pre	Post	Pre	Post	*P*^*a*^	Effect size^*b*^
Total SMI score (points)	29.3 (17.5)	15.7 (12.4)	32.4 (19.4)	32.2 (20.5)	0.001	−0.86
Vasomotor symptoms	11.0 (7.3)	5.8 (5.4)	11.6 (8.5)	13.4 (10.0)	0.003	−0.78
Physiologic symptoms	11.1 (10.4)	5.1 (5.1)	12.4 (10.8)	10.3 (8.4)	0.008	−0.48
Somatic symptoms	7.3 (4.2)	4.9 (3.4)	8.4 (4.0)	8.5 (4.3)	0.001	−0.84
SDS score (points)	40.1 (8.6)	35.8 (9.3)	42.8 (7.4)	41.6 (7.3)	0.025	−0.46

Values are mean (SD). SDS, Self-Rating Depression Scale; SMI, Simplified Menopausal Index.

^*a*^*P* value by generalized linear models, adjusted for job strain score and initial values.

^*b*^Effect size by Cohen's *d* effect sizes calculated using the difference in change between both groups (stretching group and control group).
